# Genomic Variations Underlying Speciation and Niche Specialization of *Shewanella baltica*

**DOI:** 10.1128/mSystems.00560-19

**Published:** 2019-10-15

**Authors:** Jie Deng, Jennifer M. Auchtung, Konstantinos T. Konstantinidis, Ingrid Brettar, Manfred G. Höfle, James M. Tiedje

**Affiliations:** a Shanghai Key Lab for Urban Ecological Processes and Eco-Restorations, School of Ecological and Environmental Sciences, East China Normal University, Shanghai, China; b Center for Microbial Ecology, Michigan State University, East Lansing, Michigan, USA; c Food Science and Technology Department, University of Nebraska, Lincoln, Nebraska, USA; d School of Civil and Environmental Engineering, Georgia Institute of Technology, Atlanta, Georgia, USA; e School of Biological Sciences, Georgia Institute of Technology, Atlanta, Georgia, USA; f Helmholtz Centre for Infection Research, Braunschweig, Germany; Dartmouth College

**Keywords:** *Shewanella baltica*, comparative genomics, speciation, adaptation

## Abstract

Shewanella baltica was the dominant culturable nitrate-reducing bacterium in the eutrophic and strongly stratified Baltic Sea in the 1980s, where it primarily inhabited the oxic-anoxic transition zone. The genomic structures of 46 of these isolates were investigated through comparative genomic hybridization (CGH), which revealed a gradient of genomic similarity, ranging from 65% to as high as 99%. The core genome of the S. baltica species was enriched in anaerobic respiration-associated genes. Auxiliary genes, most of which locate within a few genomic islands (GIs), were nonuniformly distributed among the isolates. Specifically, hypothetical and mobile genetic element (MGE)-associated genes dominated intraclade gene content differences, whereas gain/loss of functional genes drove gene content differences among less related strains. Among the major S. baltica clades, gene signatures related to specific redox-driven and spatial niches within the water column were identified. For instance, genes involved in anaerobic respiration of sulfur compounds may provide key adaptive advantages for clade A strains in anoxic waters where sulfur-containing electron acceptors are present. Genes involved in cell motility, in particular, a secondary flagellar biosynthesis system, may be associated with the free-living lifestyle by clade E strains. Collectively, this study revealed characteristics of genome variations present in the water column and active speciation of S. baltica strains, driven by niche partitioning and horizontal gene transfer (HGT).

**IMPORTANCE** Speciation in nature is a fundamental process driving the formation of the vast microbial diversity on Earth. In the central Baltic Sea, the long-term stratification of water led to formation of a large-scale vertical redoxcline that provided a gradient of environmental niches with respect to the availability of electron acceptors and donors. The region was home to Shewanella baltica populations, which composed the dominant culturable nitrate-reducing bacteria, particularly in the oxic-anoxic transition zone. Using the collection of S. baltica isolates as a model system, genomic variations showed contrasting gene-sharing patterns within versus among S. baltica clades and revealed genomic signatures of S. baltica clades related to redox niche specialization as well as particle association. This study provides important insights into genomic mechanisms underlying bacterial speciation within this unique natural redoxcline.

## Abstract (precis)

Speciation in nature is a fundamental process driving the formation of the vast microbial diversity on Earth. In the central Baltic Sea, the long-term stratification of water led to formation of a large-scale vertical redoxcline that provided a gradient of environmental niches with respect to the availability of electron acceptors and donors. The region was home to Shewanella baltica populations, which composed the dominant culturable nitrate-reducing bacteria, particularly in the oxic-anoxic transition zone. Using the collection of S. baltica isolates as a model system, genomic variations showed contrasting gene-sharing patterns within versus among S. baltica clades and revealed genomic signatures of S. baltica clades related to redox niche specialization as well as particle association. This study provides important insights into genomic mechanisms underlying bacterial speciation within this unique natural redoxcline.

## INTRODUCTION

A fundamental question in microbial ecology is how the enormous diversity of microorganisms on Earth developed and evolved. Over the past decades, advances in sequencing technologies have not only allowed whole-genome characterization of a range of prokaryotic organisms (1, 2) but also boosted comparative genomic studies that enabled more systematic understandings of bacterial genome evolution (3–5). One of the greatest achievements was disclosure of horizontal gene transfer (HGT), e.g., genomic islands (GIs), as one of the most important mechanisms for bacterial genomic plasticity (6–8). In addition, whole-genome-based bioinformatic algorithms were developed for more reliable assessment of phylogenetic associations among microorganisms (9–11). Nonetheless, it remains less well explored how variations in genomic content develop at different stages of bacterial diversification and how divergence accumulates and eventually leads to bacterial “species” formation. Evidence has shown that 95% in whole-genome average nucleotide identity (ANI) is a justifiable approximate for determining interspecific differentiation (10). At the intragenus level, comparative genomic studies of *Shewanella*, *Burkholderia*, *Escherichia*, and other genera have revealed different patterns of change in overall genomic profiles and in conserved genes, genes of unknown function, and genes associated with different functional categories, as well as genes associated with mobile genetic elements (MGEs) in relation to phylogenetic relatedness and ecological associations (10). However, at the intraspecific level, it has not yet been investigated how patterns of change in gene components vary across the bacterial speciation process (species formation within current named species) and whether such patterns could be used, in combination with sequence similarity, to advance bacterial species identification. In this context, a model system with an intraspecific evolutionary gradient is central and may provide important insights into the mechanisms governing the bacterial speciation process and underlying maintenance of the population structure.

In this study, we explored the genomic variations in a set of Shewanella baltica strains isolated from the stratified basin in the central Baltic Sea during 1986 to 1987 and in 1998 (12–14). These S. baltica strains were obtained through attempts to isolate nitrate-reducing bacteria in the eutrophicated Baltic Sea waters at both Gotland Deep station and Station T. Across the central Baltic, a stable, large vertical-scale redox gradient persisted between the 1970s and 1993, the formation of which was largely due to the presence of a steep halocline that prevented vertical mixing of seawater (14). S. baltica dominated all culturable nitrate reducers by over 70% and was specifically enriched in the oxic-anoxic transition zone, from the suboxic water at 80 m to completely anoxic water at 140 m (13, 15). With the high productivity in the surface water, especially over the summer, the central Baltic provided an energy-rich environment with a stable redoxcline containing a broad set of electron acceptors, including oxygen, nitrate, nitrite, and sulfur compounds, favoring the growth of respiratory generalists such as S. baltica (12).

From the original 144-strain collection, a subset of 46 S. baltica strains was selected as representatives of the major phylotypes based on random amplification of polymorphic DNA (RAPD) for detailed genetic and phenotypic profiling (16). Through Biolog assays, respiratory versatility screening, and multilocus sequence typing (MLST) using concatenated sequences of seven housekeeping genes, multiple genetically and phenotypically coherent S. baltica clades were revealed within this named species (16). Intraspecific specialization was shown to associate with nutrient availability, redox condition, and particle association as well as temporal distribution. Several of the S. baltica clades were highly uniform and deeply branched; e.g., the MLST clades A and E contained the largest numbers of strains whereas other clades were relatively smaller. Evidence suggested that clades A and E were each associated with unique niches (13, 15, 16). For instance, clade A strains were recovered only from Gotland Deep station with the presence of hydrogen sulfide in deep anoxic water. They were also favorably isolated using thiosulfate-containing medium under anaerobic culture conditions, therefore representing a group of “sulfur specialists” within S. baltica. In contrast, clade E strains were isolated only from the suboxic zone showing no presence of sulfide and favorably cultured under aerobic conditions (14). Together with other S. baltica clades, these strains formed an intraspecific gradient of genetic relatedness and provided a valuable resource for examining bacterial diversification and specialization within a short evolutionary distance.

In this study, we use this S. baltica strain collection as a model system to assay how genomic variations relate to phylogenetic relatedness and niche partitioning. Genomic sequences of four initially sequenced strains, OS185, OS195, OS223, and OS155, representing distinct clades within the species, were used to design oligonucleotide microarrays, through which the genomic contents of the 46 S. baltica strains were probed. Results from the comparative genomic hybridization (CGH) assays revealed characteristics of gene content changes at varied stages of S. baltica diversification and gathered insights on the features of the S. baltica core genome, as well as on genomic signatures of individual S. baltica clades. Altogether, our findings reveal not only evolutionary forces contributing to apparent maintenance of intraclade coherence but also the adaptive strategies of diversifying S. baltica strains at the genomic level.

## RESULTS

### The S. baltica core genome.

**FIG 1:**
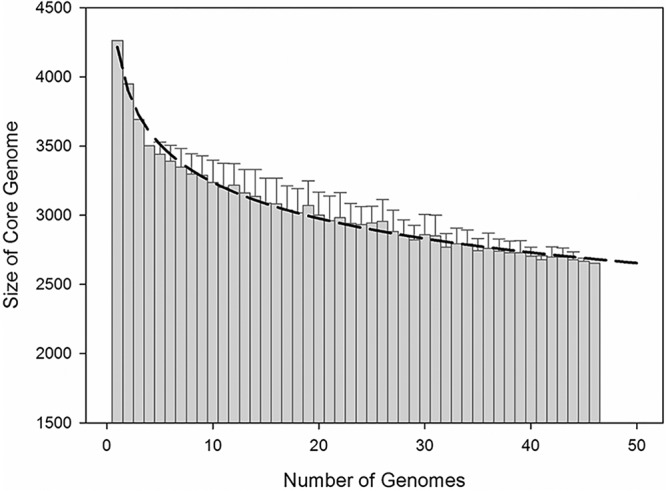
The relationship between the size of S. baltica core genome and the number of genomes analyzed. The first four genomes were OS185, OS195, OS223, and OS155. Afterward, the S. baltica genomes were randomly sampled, and the height of each bar corresponds to the average core genome size. Error bars represent the standard deviations for core genome sizes. The dashed line illustrates the power law regression model (*Y* = *aX^b^* + *c*, where *a* = 5,750, *b* = 0.08103, and *c* = −1,534) used to fit the trend line.

Among the 5,635 genes targeted on the array, 3,395 (60.2%) genes were shared among over 90% of all strains (“soft” gene core), comprising the core genome of the current S. baltica species. Fitting of the power law regression model further estimated the “hard” gene core (meaning present in all genomes) size of the entire S. baltica species to be 1,534 genes (Fig. 1). Compared to the auxiliary genes, the S. baltica core genome is enriched in genes involved in central metabolism, including those associated with transport and metabolism of coenzymes, amino acids, and nucleotides; translation; signal transduction; ribosomal structure and biogenesis; and posttranslational modification as well as energy production and conversion (see Fig. S1 in the supplemental material). Moreover, consistent with the respiratory versatility known to be characteristic of most *Shewanella* species, the S. baltica core genome is rich in genes associated with anaerobic respiration. Specifically, the S. baltica core genome includes two sets of predicted nitrate reductase systems (Shew185_0823 to -0825 and Shew185_1934 to -1938), nitrite reductase systems (Shew185_3711 and Shew185_4151 to -4154), and Na-translocating NADH-quinone reductases (Shew185_3559 to -3564 and Shew185_3433 to -3438). Previous transcriptomic assays of the type strains OS185 and OS195 have confirmed expression and differential regulation of a number of these genes under aerobic or aerobic growth conditions (17). For instance, the nitrate reductase system genes were upregulated during anaerobic growth in both OS185 and OS195, whereas the quinone reductases were aerobically upregulated (17). A sulfur reductase operon (*phsABC*, Shew185_0531 to -0533) was also anaerobically upregulated (17). Although the trimethylamine N-oxide (TMAO) reductase genes showed significant hybridization signals in all strains, physiological data suggested that strains in clade E were not able to reduce TMAO anaerobically, likely due to deletion of one nucleotide in the *torA* gene based on OS155 genome sequences (16). Compared to the genome of the type strain Shewanella oneidensis MR-1, the S. baltica core genome uniquely encodes a fumarate reductase involved in anaerobic respiration (Shew185_0614 to -0619, upregulated under anaerobic conditions in both OS185 and OS195) and an operon likely associated with inorganic ion transport during aerobic growth (Shew185_0900 to -0905, upregulated under aerobic condition in OS185 and OS195) (17). Altogether, the abundant redox-related genes found in the S. baltica core genome suggest a versatile respiratory nature of the S. baltica species, which potentially has contributed to adaptation of the S. baltica strains to the redox transition zone of the Baltic Sea.

10.1128/mSystems.00560-19.1 FIG S1 Functional distribution of S. baltica auxiliary genes and core genes. Description of COG categories is in the right panel. Genes not categorized into any COG categories are not included. Download FIG S1, TIF file, 0.3 MB. Copyright © 2019 Deng et al. 2019 Deng et al. This content is distributed under the terms of the Creative Commons Attribution 4.0 International license.

### Genomic islands.

Through mapping positions of variable genes on the chromosome of OS185, we identified 15 regions, each containing over 10 consecutive variable genes (Table 1). Most of these regions include or are adjacent to predicted mobile genetic elements (MGEs) and therefore may be acquired through horizontal gene transfer (HGT). Hence, we refer to these hypervariable regions as potential genomic islands (GIs). Among the total 816 auxiliary genes in the genome of OS185, 444 (54%) genes are located within these genomic islands. Compared to the core genome with an average GC content of 47.6%, these islands are of significantly lower GC content, suggesting codon usage bias and foreign origin of their genes. Among these islands, the majority are dominated by hypothetical and MGE-associated genes, including GI-15, containing a type IV conjugative transfer system, and GI-11, containing a number of clustered regularly interspaced short palindromic repeat (CRISPR)-related genes. There are also a few GIs, i.e., GI-8, -9, -10, -12, and -13, which contain mostly functional genes, expression of some of which was previously confirmed in strains OS185 and OS195 (17). Moreover, the patterns of gene presence/absence in these GIs are inconsistent with the overall genomic similarities based on gene presence/absence profiles (Table S1), suggesting different evolutionary paths of the GIs from the rest of the genomes. Identification of these GIs revealed the mosaic structure of S. baltica genomes. Such information has also facilitated location of potential gene-insertion hot spots in S. baltica chromosomes.

**TABLE 1:** Brief summary of genomic islands

GI	Start	End	Description	% GC content, mean ± SD	Hypo/MGE, %^*a*^	Size^*b*^
1	Shew185_0557	Shew185_0602		0.399 ± 0.068	16/4, 63	32
2	Shew185_0722	Shew185_0773		0.468 ± 0.064	27/10, 80	46
3	Shew185_1682	Shew185_1702		0.424 ± 0.045	14/5, 100	19
4	Shew185_1839	Shew185_1880		0.455 ± 0.063	24/3, 71	38
5	Shew185_1951	Shew185_1993		0.464 ± 0.056	18/5, 68	34
6	Shew185_2067	Shew185_2112		0.467 ± 0.061	24/12, 90	40
7	Shew185_2175	Shew185_2198		0.411 ± 0.034	13/1, 64	22
8	Shew185_2533	Shew185_2577	Multiple anaerobically upregulated functional genes	0.451 ± 0.06	16/0, 48	33
9	Shew185_2889	Shew185_2902	Polysaccharide biosynthesis, aerobically upregulated by OS185	0.36 ± 0.041	1/0, 7	14
10	Shew185_2971	Shew185_2982	Membrane biosynthesis- related genes	0.413 ± 0.017	4/0, 33	12
11	Shew185_3236	Shew185_3273	CRISPR-associated proteins and phage components	0.426 ± 0.071	13/2, 58	26
12	Shew185_3331	Shew185_3384	Iron complex transportation, galactose metabolism	0.441 ± 0.059	14/2, 41	39
13	Shew185_3866	Shew185_3877	Sulfite reductase *sirABIGCDJKLM*	0.473 ± 0.033	0/0, 0	11
14	Shew185_4260	Shew185_4279		0.405 ± 0.031	6/2, 57	14
15	Shew185_4383	Shew185_4457	Type F conjugative transfer system	0.437 ± 0.051	24/23, 73	64

a Number of hypothetical and MGE-associated genes and the fraction of the sum of the two in the corresponding GI.

b Number of genes represented in CGH.

10.1128/mSystems.00560-19.3 TABLE S1 Kendall correlation between gene distribution patterns of individual GIs and the overall gene distribution pattern. Download Table S1, DOCX file, 0.01 MB. Copyright © 2019 Deng et al. 2019 Deng et al. This content is distributed under the terms of the Creative Commons Attribution 4.0 International license.

### Divergence in gene content associated with phylogenetic relatedness.

**FIG 2:**
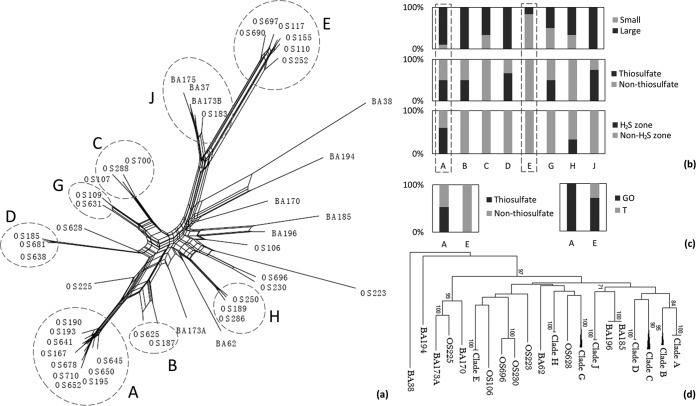
(a) Neighbor-Net analysis based on gene presence/absence profiles of the 46 S. baltica strains created by SplitsTree4 using the Jaccard distance matrix. The clades consistent with those from the MLST analysis are marked with dashed circles. (b) The percentages of strains isolated using small (<0.1 ml) or larger (≥0.1 ml) inoculum volumes, using medium with or without thiosulfate, and from H_2_S-containing or non-H_2_S-containing water zones from each clade. (c) The percentages of strains in the RAPD clades (the 144-strain collection) corresponding to MLST clades A and E using medium with or without thiosulfate and from Gotland Deep station (GO) or Station T (T). (d) Neighbor-joining tree from MLST analysis based on concatenated sequences of seven housekeeping genes. Bootstrap values (1,000 bootstrap tests) under 70% were not shown.

Based on the gene presence/absence profiles from CGH, the pairwise genomic similarities ranged from 65% to as high as nearly 99%, which formed an intraspecific genetic gradient. Distance-based Neighbor-Net analysis was performed to examine whether CGH may reveal consistent clustering patterns with those from the previous MLST analysis (18). The resulting branching patterns were consistent with the major MLST clades, while some smaller and less segregated MLST clades were disrupted (Fig. 2). The net structure suggested that a part of the gene gain/loss events was not congruent with their phylogenetic associations with the S. baltica strains. In particular, some of the small clades and individual strains branched from near the center of the net structure, indicative of impacts from HGT on genomic evolution of the S. baltica strains.

**FIG 3:**
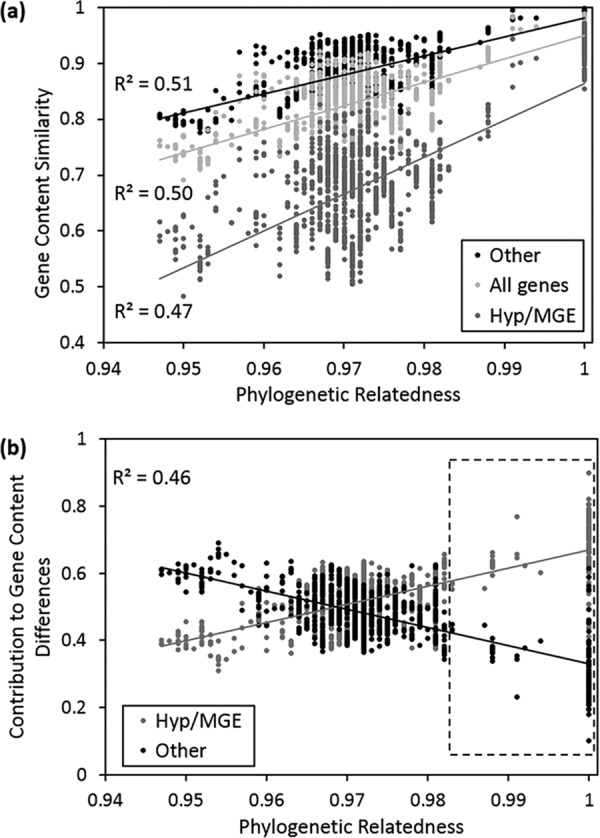
(a) Association between phylogenetic relatedness and gene content similarity based on gene presence/absence using the Jaccard distance index. (b) Differential contributions by hypothetical and MGE-associated genes versus other (functional) genes to gene content differences among strains. Within the box marked by dashed lines, the contribution from hypothetical and MGE-associated genes significantly outweighed that from other genes.

A significant association between overall gene content similarity and MLST-based phylogenetic relatedness was also revealed through analysis of the gene presence/absence profiles from CGH (Fig. 3a). We show that the extent of gene content difference is significantly higher in hypothetical and MGE-associated genes than in the other (functional) genes, suggesting that gain or loss of functional genes occurred less frequently. The differences in possession of genes associated with the 11 most abundant Clusters of Orthologous Groups (COG) functional categories among strains were further examined (Fig. S2) and revealed that genes involved in cell membrane biogenesis (M), signal transduction (T), and transcription (K) had the greatest rates of divergence. The last category, in particular, may alter patterns of transcriptional regulation, which could provide an additional mechanism in accelerating phenotypic differentiation following changes in genomic content.

10.1128/mSystems.00560-19.2 FIG S2 Gene content differences for varied COG categories across the S. baltica phylogenetic gradient. Download FIG S2, TIF file, 0.1 MB. Copyright © 2019 Deng et al. 2019 Deng et al. This content is distributed under the terms of the Creative Commons Attribution 4.0 International license.

Next, we examined the structure of gene content differences across this genetic gradient and found among very closely related strains, e.g., above 97% in MLST sequence identity, that gain/loss of hypothetical and MGE-associated genes dominated. In contrast, the contribution from gain or loss of functional genes outweighed that from hypothetical and MGE-associated genes among less closely related strains (Fig. 3b). These observations imply that functional and hypothetical or MGE-associated genes may follow different evolutionary paths and play different roles at varied stages of bacterial diversification.

### Hierarchical clustering revealed genomic signatures of S. baltica clades.

**FIG 4:**
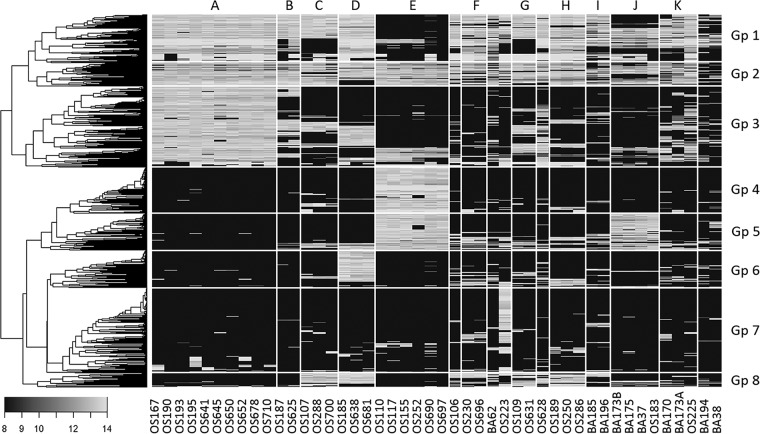
CGH heat map of all S. baltica auxiliary genes from the 46 strains. The shading key on the lower left corner illustrates logarithmized (base = 2) CGH signal intensity. Hierarchical clustering of genes revealed eight major gene groups with similar distributions among the 46 strains. The MLST genotypes are labeled on the top.

To further assess the distribution of variable genes among the S. baltica strains, hierarchical clustering was performed and yielded eight major groups with characteristic gene distribution patterns (Fig. 4). Among these gene groups, group II consisted of genes present in most of the strains. Group VII contained predominantly genes specific to OS223 and some scattered in a few S. baltica strains. Most of the other gene groups showed strong signals of distribution with respect to S. baltica clades, especially regarding clades A, E, D, and J. In a previous study, we profiled gene expression patterns of two strains, OS185 and OS195, under conditions of respiring oxygen, nitrate, and thiosulfate, which revealed genes that were differentially expressed in relation to the above redox conditions (17). Here, we further mapped the CGH profile to the gene expression profiles to discern the distribution of the genes whose presence/absence patterns were potentially associated with redox specialization.

Group I primarily features genes absent from clade E but present in most other strains. Among these, a sulfate transporter-associated cytochrome *c* gene (Shew185_0460) and a gene cluster containing a ferric uptake regulator and a pyridine nucleotide-disulfide oxidoreductase (Shew185_1391 to -1395) were upregulated in both OS185 and OS195 under the thiosulfate respiratory condition (17). Group I also includes a putative Se-containing formate dehydrogenase operon (Shew185_0099 to -0108), which, although not differentially regulated in OS185 or OS195, was induced by S. oneidensis MR-1 under the nitrate respiratory condition (19). Considering the isolation of clade E strains mostly from relatively oxic regions, the absence of these genes specifically from this clade potentially restricted them from the anoxic water zone.

Group III genes are characterized by their distribution predominantly in clade A, out of which 22% (105 genes) were differentially regulated in OS185 or OS195 (17). Specifically, this group includes both GI-10 and GI-13, with the former containing genes involved in amino sugar and nucleotide sugar metabolism (Shew185_2971 to -2982) and the latter containing a number of sulfite reductases (*sir*, Sbal195_3991 to -4002 in OS195 and Shew185_3866 to -3877 in OS185), all of which were upregulated under nitrate- or thiosulfate-respiring conditions by OS185 or OS195 (17). A number of genes involved in flagellar biosynthesis (Shew185_2942 to -2951), as well as genes for the dimethyl sulfoxide (DMSO) reductase (Sbal195_2225 to -2230) and a flavocytochrome *c* (Sbal195_0256 and -0257) that are absent from genomes of clade D, were anaerobically upregulated by OS195. Group III also includes GI-11, which encodes a number of clustered regularly interspaced short palindromic repeat (CRISPR)-associated proteins (Shew185_3236 to -3241), which may be involved in defense mechanisms against foreign genetic elements (20).

Group IV is dominated by genes nearly specific to clade E, whereas most of the group V genes are shared by clades E and J. Group IV is primarily composed of genes encoding hypothetical proteins or mobile elements, such as those within putative prophages or adjacent to transposases (Sbal_1270 to Sbal_1306 and Sbal_4384 to Sbal_4411). Within Group V, 17 genes are involved in cell wall/membrane biogenesis, and 43 genes encode a secondary flagellar biosynthesis system (Sbal_3946 to -3988). These genes are of similar organization as those from Shewanella putrefaciens CN-32 (Sputcn32_3447 to -3485), which have been shown to improve cell motility through increasing directional persistence while swimming (21, 22).

Group VI genes are primarily shared among clade D strains only. Notably, this group includes the majority of GI-8, which contains two anaerobically induced gene clusters (Shew185_2544 to -2551 and Shew185_2533 to -2538), the former likely encoding a periplasmic molecule complex including a flavocytochrome *c* and the latter encoding a putative anaerobic dehydrogenase complex and an outer membrane porin. GI-8 also includes two nitrate-inducible hypothetical genes (Shew185_3954 and -3955), as well as genes involved in cobyrinic acid *a*,*c*-diamide synthesis (Shew185_2565 and -2566), an important step in anaerobic biosynthesis of vitamin B_12_ (23). Aside from GI-8, a putative phenazine biosynthesis gene (Shew185_2184), as well as a number of aerobically upregulated polysaccharide synthesis genes (Shew185_2888 to -2903), is also shared exclusively among clade D strains.

## DISCUSSION

### Intraspecific genomic diversification among S. baltica strains.

Initial classification of the strains into S. baltica was based on DNA-DNA hybridization and 16S rRNA gene sequencing followed by phenotypic assays on selected type strains (24). Here, using CGH assays, we demonstrated pairwise genomic similarity ranging from 65% to 99% within this given species. Among the S. baltica strains, BA38, a strain isolated in 1998, was the most distantly related strain compared to all others and shared the lowest genomic similarity of 65% with OS223, obtained in 1986. However, within S. baltica clades, the genomic similarities were generally higher than 95%. The breadth of intraspecific genomic diversity revealed in this study suggests that this strain collection may provide a model system for studying bacterial diversification and speciation.

Within these S. baltica strains, a large number of strains were contained within a few phylogenetically distinct and deeply rooted clades, in particular, clades A and E (25). Expanding to the larger strain collection, 59 strains in 1986 and 54 strains in 1987 were affiliated with these two clades (14). Within each clade, the strains shared nearly identical MLST sequences and over 95% of their genomic contents (16). We hypothesize that the extent of ecological specialization was an important factor driving formation of distinct clades. As r-strategists and due to their respiratory versatility, species of *Shewanella* are generally good at responding to nutrient pulses, especially in stratified environments (26, 27). Under the impact of a large cyanobacterial bloom in the summers of 1986 and 1987, the water column of the central Baltic was supplied with increased organic material, allowing the bloom of S. baltica in both the low-oxic and anoxic water due to its versatility with respect to electron acceptor and carbon compounds. The effect of carbon availability on S. baltica was further supported by experiments showing that addition of yeast extract (1 mg/liter in C) increased the abundance of S. baltica in the low-oxic samples of 80 m and 120 m by at least 2 orders of magnitude up to a few percent of the total bacterial community (unpublished data). Therefore, formation of the two clades may be the result of ecological specialization to the local availability of electron acceptors and donors, which triggered the growth of the best-adapted S. baltica genotype, leading to a large set of strains of the same or a few clades for isolation.

Aside from clades A and E, a number of other clades were also revealed and, though containing relatively fewer strains, comprised a greater range of genomic diversity within the S. baltica species. This could be an important strategy for S. baltica to increase its survival possibilities in a water column with fluctuating conditions. With regard to this aspect, both *Shewanella* and *Burkholderia* genera are known representatives of bacteria having plastic genomes and metabolic versatilities, which enable them to adapt to complex environments (27, 28). In this study, it should be noted that CGH is a close-format genomic profiling method and could not detect genes absent from the reference strains. The overall S. baltica strain collection is expected to encompass greater genomic diversity, which whole-genome sequencing would reveal.

### Bacterial species distinction may be improved by differential distributions of functional versus hypothetical and MGE-associated genes.

Comparative genomics provides an important means to explore the genetic and genomic mechanisms underlying bacterial diversification and speciation. In particular, with this S. baltica strain collection, we were able to narrow what forces drove genomic diversification at different evolutionary stages. First, we previously showed that strains, especially in the big clades, were undergoing high rates of homologous recombination (29, 30), which could have substantially contributed to maintenance of intraclade genetic coherence. In this study, we further demonstrated that intraclade gene content differences were dominated by presence/absence of hypothetical and MGE-associated genes, whereas gain/loss of functional genes was likely inhibited. A similar trend was previously noticed through interspecific comparisons using genomes belonging to different *Shewanella s*pecies (31). This study extended the scenario into finer-scale comparisons at the intraspecific level. The results suggest that strains within the same clade tend to retain minimal functional divergence, thereby allowing limited metabolic variation, which may be key to maintenance of intraclade phenotypic and ecological consistency. Here, the transition point of 97% in sequence similarity was based on MLST analysis using the concatenation of seven housekeeping genes (16) and roughly corresponds to 80% in genomic similarity based on gene presence/absence patterns from CGH (Fig. 3a). Caution must be used while using this similarity threshold as a cutoff for distinguishing diversifying clades/strains, as considerable variations in contributions from different gene pools to overall gene content difference were still present, especially in the range of 96% to 98% in sequence identity (Fig. 3b). Nonetheless, for the S. baltica clades with higher intraclade MLST sequence similarity (98.2% to 100%, as in the boxed range with dashed line in Fig. 3b), the contribution from hypothetical and MGE-associated genes significantly outweighed that from functional genes (*P < *0.0001). Taken together, both the genetic and ecological evidence suggests that the big and deep-branching S. baltica clades at least, i.e., clades A and E, justifiably represent the very basic unit of bacterial diversity. In other words, both clades have differentiated/speciated enough to become distinct units that could maintain their own genetic integrity, in other words, different “species,” within the current species of S. baltica.

Finally, these observations also raise the question of whether these hypothetical and MGE-associated genes are neutral or simply “junk” DNAs in S. baltica genomes. At the current stage, we still lack bioinformatic tools to effectively detect “useless” sequences in bacterial genomes. Nonetheless, in agreement with previous studies (10, 31), our results suggest that hypothetical and MGE-related genes should be treated differently in cross-genome comparisons, as these genes may be of different evolutionary trajectories. In addition, some GIs identified from this study are dominated by hypothetical and MGE-associated genes, whereas others contain mostly functional genes with none or only a few MGE-associated genes. Although gene presence/absence patterns of all these GIs were weakly associated with overall genomic similarities, we believe that it is still worthwhile to look further into whether evolution of these two types of GIs in bacterial genomes is governed by different mechanisms through more sequence-based comparisons.

### Diversification under impacts from niche partitioning.

Niche partitioning leads to selective pressure that drives sequence evolution, which eventually results in physiological differentiation. In this study, we observed genomic variations in relation to niches associated with different redox conditions and spatial distributions of the S. baltica strains. For instance, specialization under anaerobic niche environments at the genomic level was exemplified by possession of the group III auxiliary genes by clade A strains, particularly those responsible for respiration of sulfur compounds, e.g., the sulfite reductases, the DMSO reductase, the sulfite dehydrogenase (Sbal195_0006 to -0009), and the thiosulfate-inducible cytochrome *c* (Shew185_0460). Upregulation of these genes under anaerobic thiosulfate- or nitrate-respiring conditions was confirmed through comparative transcriptomic analyses of the type strains OS185 and OS195 (17). These genomic signatures are consistent with the respiratory versatility of clade A strains and their isolation preferably from thiosulfate-supplemented anaerobic medium, which together suggest that clade A strains may be better adapted to the anoxic water zone where alternative electron acceptors, especially sulfur compounds, are available.

Spatial distribution, i.e., being particle attached versus free living, provides additional selective pressure causing intraspecific diversification. Particles in offshore marine environments are mostly dominated by phytoplankton-derived organic matter, therefore forming individual nutrient-rich “islands” in the relatively nutrient-poor water column (32). Many studies have reported significant differences between particle-attached and free-living bacterial communities. The fact that the majority of the S. baltica strains were associated with particle-attached conditions implies that nitrate reduction may be partly driven by consumption of organic carbon associated with these particles in the eutrophied Baltic Sea. In contrast, clade E strains, most frequently recovered from the lowest dilution steps and putatively representing a free-living lifestyle with high abundance *in situ* (14, 16), may couple nitrate reduction with turnover of different types of organic matter. Compared to other S. baltica strains, clade E strains were less versatile in carbon utilization capabilities (16), consistent with the fact that nutrient levels are lower in the water column than on particles. In addition, clade E was the only genotype recovered in both the years 1986 and 1987 and from both Gotland Deep and Station T, which were separated by over 200 km. An additional finding on genes related to the free-living lifestyle was the presence of a secondary flagellar biosynthesis system in the genomes of both clades E and J. A previous study using S. putrefaciens CN-32 suggested that the secondary flagellar system increased cell motility and radical spreading in soft agar medium (21). The expression of the secondary system occurred under the planktonic condition in complex medium and resulted in formation of up to three additional flagella extending from random positions around the cell. In the case of S. baltica, expression of the secondary flagellar system under (temporary) nutrient-rich conditions may allow a subpopulation to be better equipped for efficient spreading and colonization into new habitats. Therefore, this gene set could be a key feature associated with the free-living style of clade E strains and might have contributed to successful propagation despite their lack of metabolic flexibility.

### Gaining of functions through HGT by the S. baltica strains.

HGT is an important means of gene acquisition in bacterial genomes (6, 33). Comparative genomic studies have revealed HGT of genes related to pathogenicity (34, 35), antibiotic resistance (36), heavy metal resistance (37), and nitrogen fixation (38–40). Some of these horizontally acquired functions have contributed key adaptive advantages for the bacterial hosts in their specific environments. Among the putative GIs identified in this study, most were likely acquired horizontally due to skewed GC content as well as the presence of integrases or other MGE-related genes. Some of the functional GIs contain genes that are clearly ecologically relevant: for instance, those in GI-8 and -13 that associate with anaerobic respiration and were confirmed by gene expression assays and genes of GI-12 likely involved in iron complex transport and galactose metabolism. Notably, GI-11 contains six CRISPR-associated proteins (Shew185_3236 to -3241). A previous report suggested exchange of CRISPR-associated proteins among *Shewanella* genomes through HGT mediated by megaplasmids (41). In this case, the downstream region of CRISPR-associated genes in OS185 and OS195 includes a putative phage repressor (Shew185_3262) and a phage integrase (Shew185_3273). Although HGT of CRISPR-associated genes by phages is rarely reported, conserved synteny of upstream genes in sequenced S. baltica genomes further supports insertion of the CRISPR genes through HGT (41).

In addition to the relatively large islands, a number of other genes and operons are also flanked by MGEs, including those genes that are likely ecologically relevant. For instance, the DMSO reductase operon is downstream of a phage integrase (Sbal195_2224) in OS195, and the secondary flagellar biosynthesis system in clades E and J is close to a gene encoding an integrase catalytic unit (Sbal_3944) in OS155. These MGE-associated genes, together with the putative GIs, likely contributed to the genomic plasticity within the S. baltica species and have endowed the S. baltica strains with accessory capabilities that may be important for the persistence of S. baltica in the Baltic Sea over decades.

### Summary.

Through CGH analyses, this study revealed a gradient of intraspecific genomic variation within the S. baltica strain collection. Among closely related strains within individual S. baltica clades, genomic contents were highly similar and gene content differences were dominated by the presence/absence of hypothetical and MGE-related genes. In contrast, among less phylogenetically related strains, genomic variations were increasingly attributed to functional genes. Accordingly, genomic signatures, i.e., characteristic distributions of functional genes, were identified and associated with redox specialization and spatial distribution of the major S. baltica clades. Altogether, these results generated insights into differences in evolutionary mechanisms along the bacterial speciation process in a spatially stable redox (chemical) gradient and, more generally, provide implications for bacterial species distinction using comparative genomic tools.

## MATERIALS AND METHODS

### Strain description and DNA extraction.

S. baltica strains used in this study were described in reference 16. Details regarding array design were described in reference 17. Briefly, probes were 44 to 48 nucleotides long and were designed from the genomic sequences of four reference S. baltica strains, at the sequence order of OS185 > OS195 > OS223 > OS155 when homologous gene sequences diverged. Up to seven probes were designed for each gene, and a total of 30,000 probes targeting the S. baltica genomes were designed, with an additional 720 probes as negative controls. The strains were grown in LB medium (Acumedia, Lansing, MI, USA) at 22°C, and DNA was extracted using a modified cetyltrimethylammonium bromide (CTAB) genomic extraction protocol (42).

### Microarray hybridization and data processing.

For DNA-DNA hybridization assays, DNA was first sonicated to produce fragments of less than 3 kbp in size and was then labeled with the fluorescent Cy5 dye by incorporation of amino-allyl-dUTP through extension from random primers using Escherichia coli DNA polymerase Klenow fragment I (Invitrogen, Grand Island, NY), followed by addition of amine-reactive Cy5. Cy5-labeled DNA samples were hybridized to the microarrays at 50°C for 18 h before being washed and scanned using an Axon GenePix 4000B scanner (one-channel hybridization). More details of the hybridization protocol were described in reference 29. For array data processing and normalization, mean signal intensity from the negative-control probes was first subtracted from the signal intensities of all spots. Signals of individual genes were calculated as the median value for normalized signals from all probes designed for that gene. A cutoff value was used to determine the presence/absence of the genes. The cutoff value was optimized to achieve the minimum false-discovery rates (FDRs) based on hybridization profiles of DNA from the four reference genomes—OS185, OS195, OS223, and OS155. Specifically, the optimized cutoff was 850 signal units, at which the FDR was 2.2%. After sequencing of five additional S. baltica genomes in a later year (43), the FDR was reexamined to determine if there was bias toward genomes not represented on the array. As a result, the FDR ranged from 2% (OS183) to 4.4% (BA175), which was slightly higher than, yet still comparable to, those estimated based on the reference genomes, i.e., 1.6% (OS223) to 2.8% (OS195).

### Data availability.

All microarray profiles were uploaded to the GEO repository with accession number GSE123978.

10.1128/mSystems.00560-19.4 TABLE S2 Number of probes designed from each reference strain. Download Table S2, DOCX file, 0.01 MB. Copyright © 2019 Deng et al. 2019 Deng et al. This content is distributed under the terms of the Creative Commons Attribution 4.0 International license.

## References

[1] Kyrpides NC 2009 Fifteen years of microbial genomics: meeting the challenges and fulfilling the dream. Nat Biotechnol 27:627–632. doi:10.1038/nbt.1552. 19587669

[2] Mukherjee S, Seshadri R, Varghese NJ, Eloe-Fadrosh EA, Meier-Kolthoff JP, Göker M, Coates RC, Hadjithomas M, Pavlopoulos GA, Paez-Espino D, Yoshikuni Y, Visel A, Whitman WB, Garrity GM, Eisen JA, Hugenholtz P, Pati A, Ivanova NN, Woyke T, Klenk H-P, Kyrpides NC 2017 1,003 reference genomes of bacterial and archaeal isolates expand coverage of the tree of life. Nat Biotechnol 35:676–683. doi:10.1038/nbt.3886. 28604660

[3] Eisen JA, Fraser CM 2003 Phylogenomics: intersection of evolution and genomics. Science 300:1706–1707. doi:10.1126/science.1086292. 12805538

[4] Wu D, Hugenholtz P, Mavromatis K, Pukall R, Dalin E, Ivanova NN, Kunin V, Goodwin L, Wu M, Tindall BJ, Hooper SD, Pati A, Lykidis A, Spring S, Anderson IJ, D’haeseleer P, Zemla A, Singer M, Lapidus A, Nolan M, Copeland A, Han C, Chen F, Cheng J-F, Lucas S, Kerfeld C, Lang E, Gronow S, Chain P, Bruce D, Rubin EM, Kyrpides NC, Klenk H-P, Eisen JA 2009 A phylogeny-driven genomic encyclopaedia of Bacteria and Archaea. Nature 462:1056–1060. doi:10.1038/nature08656. 20033048 PMC3073058

[5] Luo C, Walk ST, Gordon DM, Feldgarden M, Tiedje JM, Konstantinidis KT 2011 Genome sequencing of environmental *Escherichia coli* expands understanding of the ecology and speciation of the model bacterial species. Proc Natl Acad Sci U S A 108:7200–7205. doi:10.1073/pnas.1015622108. 21482770 PMC3084108

[6] Dobrindt U, Hochhut B, Hentschel U, Hacker J 2004 Genomic islands in pathogenic and environmental microorganisms. Nat Rev Microbiol 2:414–424. doi:10.1038/nrmicro884. 15100694

[7] Gogarten JP, Townsend JP 2005 Horizontal gene transfer, genome innovation and evolution. Nat Rev Microbiol 3:679–687. doi:10.1038/nrmicro1204. 16138096

[8] Gal-Mor O, Finlay BB 2006 Pathogenicity islands: a molecular toolbox for bacterial virulence. Cell Microbiol 8:1707–1719. doi:10.1111/j.1462-5822.2006.00794.x. 16939533

[9] Varghese NJ, Mukherjee S, Ivanova N, Konstantinidis KT, Mavrommatis K, Kyrpides NC, Pati A 2015 Microbial species delineation using whole genome sequences. Nucleic Acids Res 43:6761–6771. doi:10.1093/nar/gkv657. 26150420 PMC4538840

[10] Konstantinidis KT, Tiedje JM 2005 Genomic insights that advance the species definition for prokaryotes. Proc Natl Acad Sci U S A 102:2567–2572. doi:10.1073/pnas.0409727102. 15701695 PMC549018

[11] Rodriguez-R LM, Gunturu S, Harvey WT, Rosselló-Mora R, Tiedje JM, Cole JR, Konstantinidis KT 2018 The Microbial Genomes Atlas (MiGA) webserver: taxonomic and gene diversity analysis of Archaea and Bacteria at the whole genome level. Nucleic Acids Res 46:W282–W288. doi:10.1093/nar/gky467. 29905870 PMC6031002

[12] Brettar I, Höfle MG 1993 Nitrous oxide producing heterotrophic bacteria from the water column of the central Baltic: abundance and molecular identification. Mar Ecol Prog Ser 94:253–265. doi:10.3354/meps094253.

[13] Brettar I, Moore ERB, Höfle MG 2001 Phylogeny and abundance of novel denitrifying bacteria isolated from the water column of the central Baltic Sea. Microb Ecol 42:295–305. doi:10.1007/s00248-001-0011-2. 12024255

[14] Ziemke F, Brettar I, Höfle MG 1997 Stability and diversity of the genetic structure of a *Shewanella putrefaciens* population in the water column of the central Baltic. Aquat Microb Ecol 13:63–74. doi:10.3354/ame013063.

[15] Höfle MG, Brettar I 1996 Genotyping of heterotrophic bacteria from the central Baltic Sea by use of low-molecular-weight RNA profiles. Appl Environ Microbiol 62:1383–1390. 16535296 10.1128/aem.62.4.1383-1390.1996 PMC1388834

[16] Deng J, Brettar I, Luo C, Auchtung J, Konstantinidis KT, Rodrigues JLM, Höfle M, Tiedje JM 2014 Stability, genotypic and phenotypic diversity of *Shewanella baltica* in the redox transition zone of the Baltic Sea. Environ Microbiol 16:1854–1866. doi:10.1111/1462-2920.12344. 24286373

[17] Deng J, Auchtung JM, Konstantinidis KT, Caro-Quintero A, Brettar I, Höfle M, Tiedje JM 2018 Divergence in gene regulation contributes to sympatric speciation of *Shewanella baltica* strains. Appl Environ Microbiol 84:e02015-17. doi:10.1128/AEM.02015-17. 29222101 PMC5795076

[18] Huson DH, Bryant D 2006 Application of phylogenetic networks in evolutionary studies. Mol Biol Evol 23:254–267. doi:10.1093/molbev/msj030. 16221896

[19] Beliaev AS, Klingeman DM, Klappenbach JA, Wu L, Romine MF, Tiedje JM, Nealson KH, Fredrickson JK, Zhou J 2005 Global transcriptome analysis of *Shewanella oneidensis* MR-1 exposed to different terminal electron acceptors. J Bacteriol 187:7138–7145. doi:10.1128/JB.187.20.7138-7145.2005. 16199584 PMC1251602

[20] Sorek R, Kunin V, Hugenholtz P 2008 CRISPR—a widespread system that provides acquired resistance against phages in bacteria and archaea. Nat Rev Microbiol 6:181–186. doi:10.1038/nrmicro1793. 18157154

[21] Bubendorfer S, Held S, Windel N, Paulick A, Klingl A, Thormann KM 2012 Specificity of motor components in the dual flagellar system of *Shewanella putrefaciens* CN-32. Mol Microbiol 83:335–350. doi:10.1111/j.1365-2958.2011.07934.x. 22151089

[22] Bubendorfer S, Koltai M, Rossmann F, Sourjik V, Thormann KM 2014 Secondary bacterial flagellar system improves bacterial spreading by increasing the directional persistence of swimming. Proc Natl Acad Sci U S A 111:11485–11490. doi:10.1073/pnas.1405820111. 25049414 PMC4128160

[23] Fresquet V, Williams L, Raushel FM 2004 Mechanism of cobyrinic acid *a,c*-diamide synthetase from *Salmonella typhimurium* LT2. Biochemistry 43:10619–10627. doi:10.1021/bi048972x. 15311923

[24] Ziemke F, Höfle MG, Lalucat J, Rosselló-Mora R 1998 Reclassification of *Shewanella putrefaciens* Owen’s genomic group II as *Shewanella baltica* sp. nov. Int J Syst Bacteriol 48:179–186. doi:10.1099/00207713-48-1-179. 9542087

[25] Hanage WP, Fraser C, Spratt BG 2006 Sequences, sequence clusters and bacterial species. Philos Trans R Soc Lond B Biol Sci 361:1917–1927. doi:10.1098/rstb.2006.1917. 17062411 PMC1764932

[26] Hau HH, Gralnick JA 2007 Ecology and biotechnology of the genus *Shewanella*. Annu Rev Microbiol 61:237–258. doi:10.1146/annurev.micro.61.080706.093257. 18035608

[27] Fredrickson JK, Romine MF, Beliaev AS, Auchtung JM, Driscoll ME, Gardner TS, Nealson KH, Osterman AL, Pinchuk G, Reed JL, Rodionov DA, Rodrigues JLM, Saffarini DA, Serres MH, Spormann AM, Zhulin IB, Tiedje JM 2008 Towards environmental systems biology of Shewanella. Nat Rev Microbiol 6:592–603. doi:10.1038/nrmicro1947. 18604222

[28] Compant S, Nowak J, Coenye T, Clément C, Ait Barka E 2008 Diversity and occurrence of *Burkholderia spp.* in the natural environment. FEMS Microbiol Rev 32:607–626. doi:10.1111/j.1574-6976.2008.00113.x. 18422616

[29] Caro-Quintero A, Deng J, Auchtung J, Brettar I, Höfle MG, Klappenbach J, Konstantinidis KT 2011 Unprecedented levels of horizontal gene transfer among spatially co-occurring *Shewanella bacteria* from the Baltic Sea. ISME J 5:131–140. doi:10.1038/ismej.2010.93. 20596068 PMC3105679

[30] Caro-Quintero A, Auchtung J, Deng J, Brettar I, Höfle M, Tiedje JM, Konstantinidis KT 2012 Genome sequencing of five *Shewanella baltica* strains recovered from the oxic-anoxic interface of the Baltic Sea. J Bacteriol 194:1236. doi:10.1128/JB.06468-11. 22328742 PMC3294791

[31] Konstantinidis KT, Serres MH, Romine MF, Rodrigues JLM, Auchtung J, McCue L-A, Lipton MS, Obraztsova A, Giometti CS, Nealson KH, Fredrickson JK, Tiedje JM 2009 Comparative systems biology across an evolutionary gradient within the *Shewanella* genus. Proc Natl Acad Sci U S A 106:15909–15914. doi:10.1073/pnas.0902000106. 19805231 PMC2747217

[32] Watanabe K, Kuwae T 2015 How organic carbon derived from multiple sources contributes to carbon sequestration processes in a shallow coastal system? Glob Chang Biol 21:2612–2623. doi:10.1111/gcb.12924. 25880367 PMC4676932

[33] Frost LS, Leplae R, Summers AO, Toussaint A 2005 Mobile genetic elements: the agents of open source evolution. Nat Rev Microbiol 3:722–732. doi:10.1038/nrmicro1235. 16138100

[34] Nisan I, Wolff C, Hanski E, Rosenshine I 1998 Interaction of enteropathogenic *Escherichia coli* with host epithelial cells. Folia Microbiol (Praha) 43:247–252. doi:10.1007/bf02818609. 9717251

[35] Malott RJ, Baldwin A, Mahenthiralingam E, Sokol PA 2005 Characterization of the *cciIR* quorum-sensing system in *Burkholderia cenocepacia*. Infect Immun 73:4982–4992. doi:10.1128/IAI.73.8.4982-4992.2005. 16041013 PMC1201253

[36] Turner SA, Luck SN, Sakellaris H, Rajakumar K, Adler B 2001 Nested deletions of the SRL pathogenicity island of *Shigella flexneri* 2a. J Bacteriol 183:5535–5543. doi:10.1128/JB.183.19.5535-5543.2001. 11544215 PMC95444

[37] Osborn AM, Bruce KD, Ritchie DA, Strike P 1996 The mercury resistance operon of the IncJ plasmid pMERPH exhibits structural and regulatory divergence from other Gram-negative *mer* operons. Microbiology 142:337–345. doi:10.1099/13500872-142-2-337. 8932707

[38] Baar C, Eppinger M, Raddatz G, Simon J, Lanz C, Klimmek O, Nandakumar R, Gross R, Rosinus A, Keller H, Jagtap P, Linke B, Meyer F, Lederer H, Schuster SC 2003 Complete genome sequence and analysis of *Wolinella succinogenes*. Proc Natl Acad Sci U S A 100:11690–11695. doi:10.1073/pnas.1932838100. 14500908 PMC208819

[39] Kaneko T, Nakamura Y, Sato S, Asamizu E, Kato T, Sasamoto S, Watanabe A, Idesawa K, Ishikawa A, Kawashima K, Kimura T, Kishida Y, Kiyokawa C, Kohara M, Matsumoto M, Matsuno A, Mochizuki Y, Nakayama S, Nakazaki N, Shimpo S, Sugimoto M, Takeuchi C, Yamada M, Tabata S 2000 Complete genome structure of the nitrogen-fixing symbiotic bacterium *Mesorhizobium loti*. DNA Res 7:331–338. doi:10.1093/dnares/7.6.331. 11214968

[40] Kaneko T, Nakamura Y, Sato S, Minamisawa K, Uchiumi T, Sasamoto S, Watanabe A, Idesawa K, Iriguchi M, Kawashima K, Kohara M, Matsumoto M, Shimpo S, Tsuruoka H, Wada T, Yamada M, Tabata S 2002 Complete genomic sequence of nitrogen-fixing symbiotic bacterium *Bradyrhizobium japonicum* USDA110. DNA Res 9:189–197. doi:10.1093/dnares/9.6.189. 12597275

[41] Godde J, Bickerton A 2006 The repetitive DNA elements called CRISPRs and their associated genes: evidence of horizontal transfer among prokaryotes. J Mol Evol 62:718–729. doi:10.1007/s00239-005-0223-z. 16612537

[42] Feil WS, Feil H, Copeland A 2012 Bacterial genomic DNA isolation using CTAB. DOE Joint Genome Institute, Walnut Creek, CA.

[43] Schicklberger M, Bücking C, Schuetz B, Heide H, Gescher J 2011 Involvement of the *Shewanella oneidensis* decaheme cytochrome MtrA in the periplasmic stability of the beta-barrel protein MtrB. Appl Environ Microbiol 77:1520–1523. doi:10.1128/AEM.01201-10. 21169449 PMC3067208

