# Supplementary figures and images for: Genomic Variations Underlying Speciation and Niche Specialization of Shewanella baltica
Source: mSystems. 2019 Oct 15;4(5):e00560-19. doi: 10.1128/mSystems.00560-19 (PMC6794122; doi:10.1128/mSystems.00560-19)

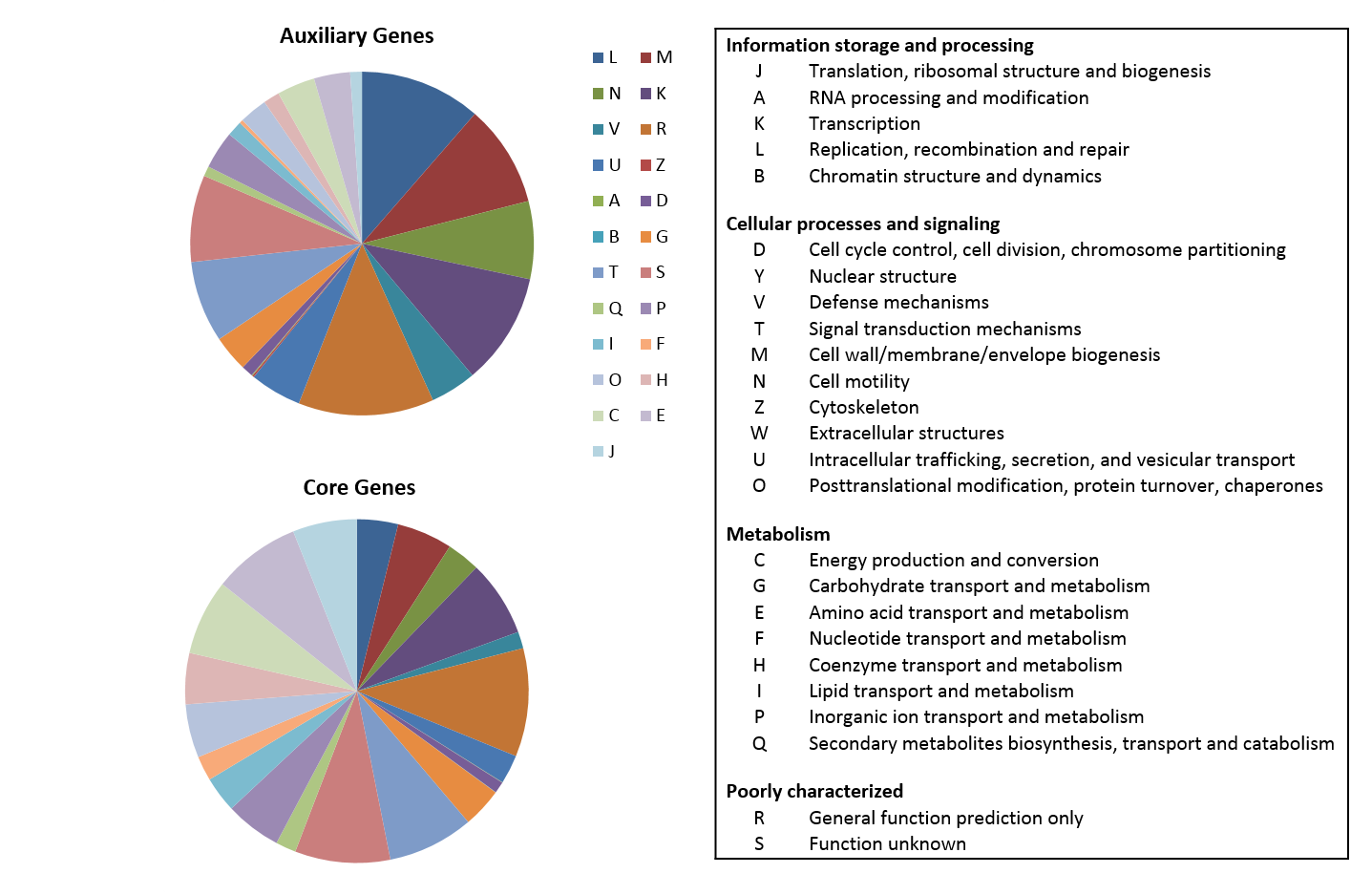

Supplement: FIG S1 [file mSystems.00560-19-sf001.tif]

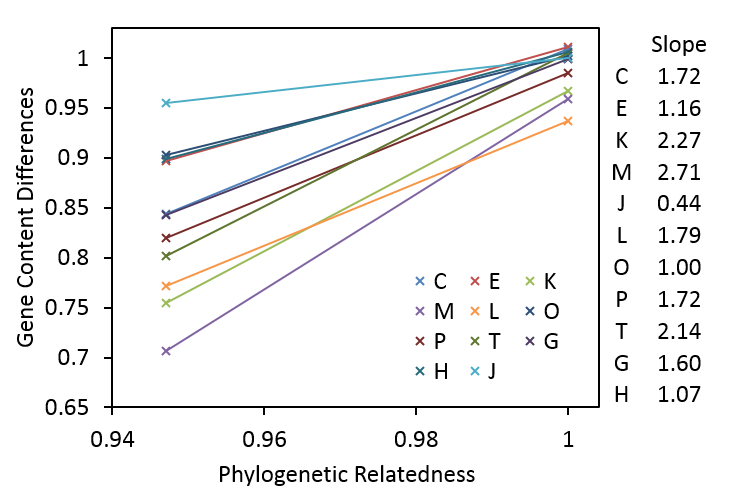

Supplement: FIG S2 [file mSystems.00560-19-sf002.tif]
