# Supplementary material for: Genomic Variations Underlying Speciation and Niche Specialization of Shewanella baltica
Source: mSystems. 2019 Oct 15;4(5):e00560-19. doi: 10.1128/mSystems.00560-19 (PMC6794122; doi:10.1128/mSystems.00560-19)
Supplement: TABLE S1 [file mSystems.00560-19-st001.docx]

**Table S1. Kendall correlation between gene distribution patterns of individual GIs with the overall gene distribution pattern**

| **Genomic Island** | ***tau*** | ***P* value** |
| --- | --- | --- |
| GI-1 | 0.034 | 0.1460 |
| GI-2 | 0.076 | 0.0004 |
| GI-3 | 0.024 | 0.2817 |
| GI-4 | 0.099 | 0.0000 |
| GI-5 | 0.088 | 0.0000 |
| GI-6 | 0.034 | 0.1425 |
| GI-7 | -0.032 | 0.1574 |
| GI-8 | 0.085 | 0.0001 |
| GI-9 | 0.057 | 0.0209 |
| GI-10 | 0.031 | 0.2170 |
| GI-11 | 0.088 | 0.0000 |
| GI-12 | 0.072 | 0.0007 |
| GI-13 | -0.035 | 0.1254 |
| GI-14 | 0.150 | 0.0000 |
| GI-15 | -0.016 | 0.4529 |
| Other functional genes | 0.859 | 0.0000 |
