# Supplementary material for: Genomic Variations Underlying Speciation and Niche Specialization of Shewanella baltica
Source: mSystems. 2019 Oct 15;4(5):e00560-19. doi: 10.1128/mSystems.00560-19 (PMC6794122; doi:10.1128/mSystems.00560-19)
Supplement: TABLE S2 [file mSystems.00560-19-st002.docx]

**Table S2.** **Number of probes designed from each reference strain**

|  | Number of Probes | Number of Genes |
| --- | --- | --- |
| OS185 | 23192 | 4228 |
| OS195 | 2459 | 642 |
| OS223 | 2112 | 532 |
| OS155 | 2236 | 580 |
| Total | 30000 | 5982 |
